# Logistics for expanding heart transplantation from donation after circulatory death using normothermic regional perfusion

**DOI:** 10.1016/j.xjtc.2022.01.014

**Published:** 2022-01-21

**Authors:** Nader Moazami, Deane Smith, Aubrey Galloway

**Affiliations:** Department of Cardiothoracic Surgery, New York University (NYU) Langone Health, New York, NY

**Keywords:** DCD, heart transplantation, NRP, normothermic regional perfusion

**Figure:**
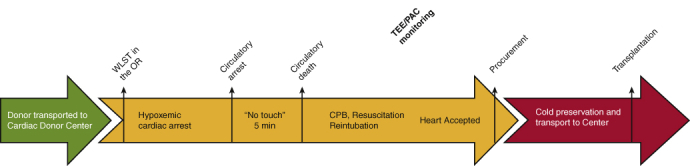
The patient is transported to a designated donor center for resuscitation and evaluation.

**Central Message**

Heart-donation centers should be established to conduct normothermic regional perfusion (NRP) from donation after circulatory death (DCD).

In recent years, there has been a growing interest in the use of donation after circulatory death (DCD) hearts for transplantation.^1^ Successful reports from Australia and the United Kingdom ignited interest in the United States to re-evaluate these approaches to DCD donors.^2^, ^3^, ^4^ Estimates suggest that heart transplantation in the United States could expand by as much as 20% if DCD programs could be successfully launched. If these estimates are true, successful strategies to use this previously untapped source of donor hearts could potentially initiate the largest expansion in heart transplantation in recent years. It is, therefore imperative that clinicians in the United States understand and evaluate this new opportunity to increase the number of heart transplants, shorten time on the waitlist, and make a significant reduction in waitlist mortality.

Recently, 2 different approaches have enabled successful transplantation using DCD hearts. The first reports used ex vivo machine perfusion (MP) to resuscitate donor hearts after a period of circulatory arrest and cardiac death. This method largely follows the established standards of organ donation and procurement used for abdominal organs from DCD donors. The primary change is that, after declaration of circulatory death and sternotomy, 1 to 1.5 L of the donor's blood is aspirated to prime the ex vivo circuit. Resuscitation of the heart is accomplished by MP using oxygenated blood perfusing the unloaded beating heart, similar to a Langendorff heart preparation. The suitability of the heart for transplantation is assessed primarily by evaluating the arteriovenous difference in lactate levels. Although the initial results with MP have been encouraging, there has been some concern over the greater need of mechanical circulatory support for primary graft dysfunction, which leads to questions as to whether arteriovenous lactate difference is a valid marker for the degree of cardiac injury and is predictive of posttransplant cardiac function. Inevitably, there will also be questions of how to address the substantially greater extra cost associated with MP. A large multicenter trial using the MP approach has been completed, although the results have not been published.

The second approach, normothermic regional perfusion (NRP), was first adopted and reported by the UK group. This approach was an adaptation of a method that was previously used successfully for abdominal organ procurement, now expanded to include thoracic organs. In the UK group's technique, the re-establishment of circulation of blood to the brain is prevented by ligation of the cerebral vessels before reinitiation of circulatory support with extracorporeal membrane oxygenation (ECMO) for heart recovery. For distant transplantation, the recovered hearts are transported by placing them on MP, or in 3 instances with standard cold storage. The UK group has reported experiences with both MP and NRP approaches for DCD heart transplant, with the NRP experience being primarily limited due to technical restrictions in the number of hospitals that can perform it. Their most recent 5-year report, however, suggests that outcomes using NRP-procured hearts are better than those achieved with MP.^2^

## Use of NRP Procurement for Heart Transplantation in the United States: The Initial NYU Experience

In January of 2020, our group performed the first NRP DCD heart transplant in the United States, using standard cardiopulmonary bypass (CPB) for resuscitation after circulatory death. Given the novelty and lack of experience with this approach, we opted to have the potential donors transported to our hospital's intensive care unit for evaluation. Consent was obtained independently by the coordinating local organ procurement organization (OPO) in our region (LiveOnNY). Institutional review board approval was obtained on November 25, 2019 (i19-01664). Patients consented to the publication of data.

**Figure 1:**
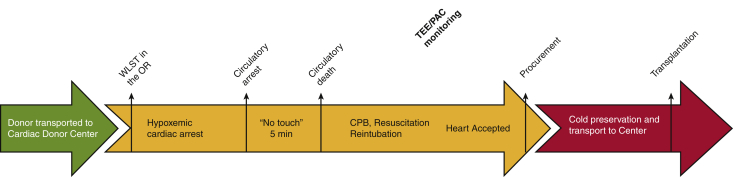
Depiction of a potential process for donation after circulatory death heart donation. *WLST*, Withdrawal of life support; *OR*, operating room; *TEE*, transesophageal echocardiography; *PAC*, pulmonary artery catheter, *CPB*, cardiopulmonary bypass.

At the appropriate time, the donor and the recipient were transferred to separate and proximate operating rooms. For the donor, heparin was administered 3 minutes before withdrawal of life support. After declaration of circulatory death and reconfirmation 5 minutes later (standoff period), a sternotomy was performed and the cerebral vessels were ligated. This was followed by standard central cannulation and normothermic CPB. The advantages afforded by CPB include the ability to place a left ventricular vent for complete cardiac decompression and unloaded myocardial reperfusion. In addition, CPB allows rapid correction of the hyperkalemia and profound metabolic acidosis that accompanies circulatory death. Finally, this approach allows the team to volume reload the heart and wean off CPB for a complete assessment of cardiac function under normal physiologic conditions prior to accepting the heart. Hemodynamic monitoring of intracardiac filling pressures and cardiac output is complemented by transesophageal echocardiographic evaluation of cardiac wall motion and contractility (Figure 1).

We recently reported our experience with 8 DCD-NRP heart transplants (including 1 heart–lung transplant) using this approach, with 100% survival, no significant primary cardiac graft dysfunction, and no need for mechanical circulatory support for cardiac dysfunction in the per-operative period.^5^ Although reperfusion and recirculation can be achieved by using closed systems such as ECMO, the advantages offered with open, traditional CPB and unloaded cardiac reperfusion are unique. Previous work has clearly demonstrated the protective impact of reperfusion of the ischemic heart in an unloaded state, which results in less reperfusion injury, improved myocardial salvage, and improved cardiac functional recovery.^6^ In addition, the ability to more rapidly correct hyperkalemia and acidosis reduces the damaging effect of these metabolic derangements on the recovering heart.

## Expanding the NRP Technique for Cardiac Allograft Recovery and Heart Transplantation: A Proposed Multicenter Approach

Although certainly both the MP and NRP approaches for the use of DCD hearts for heart transplantation have been successful, several of the aforementioned advantages of the NRP technique suggest to us that this is the more promising method. For safety reasons, we initially limited our approach geographically to our donor-specific area (DSA) to allow for colocalization of the donor and recipient. Transfer of the potential donors to our center ensured compliance with the protocol and allowed involvement of a specialized transplant intensive care unit team that was familiar with donor management and withdrawal of life support strategies. Additional advantages included having an experienced cardiac surgical operating team consisting of anesthesiologist, perfusionist, nursing, and other experienced operating room personnel to ensure the smooth conduct of a complex operative and procurement strategy. Having experienced surgeons perform the donor procedure was also extremely valuable in achieving optimal outcomes. Finally, highly trained cardiac anesthesiologists, transplant cardiologists, and transplant surgeons, all with extensive experience, performed the evaluation of the heart after resuscitation on CPB and before final acceptance.

Is this all necessary for the success of DCD heart transplantation using NRP? We believe that at the present time, the answer to this question is a resounding yes, at least until further experience suggests otherwise. For all the aforementioned reasons, we believe that these transplants should be concentrated in specialized centers accustomed to cardiac surgery. There is certainly some precedent for this strategy. Transfer of potential donors to facilities other than the initial hospital has been an established practice for close to 2 decades. In fact, the number of hospital-independent facilities for multiorgan procurement has been increasing over the past several years, and now some hospitals have initiated a similar approach.

Based on this success, it is very likely that within the DSA of each OPO, 1 or 2 cardiac transplant centers could be selected to serve as a designated DCD heart center. With this model, once a recipient who is willing to accept the heart is identified in the United Network for Organ Sharing database, the potential donor would be transferred to the designated DCD heart center in that DSA. At this center, a dedicated cardiac transplant surgical team would conduct the reperfusion, assessment for suitability, and eventual procurement of the heart as outlined previously. The heart would then be preserved and transported to the distant recipient center using standard cold storage. In our view, this model would allow the best-available resources in the DSA of each OPO to resuscitate and evaluate hearts from DCD donors for eventual successful heart transplantation at a distant site.

Several limitations and questions can occur with this model. For example, what if the family does not agree to the transfer to a DCD-designated center because of concerns related to distance? Who bears the actual cost of the transfers, and what happens in the potential scenarios in which the donor does not progress to circulatory arrest?

Since the United Network for Organ Sharing does not regulate the methodology or location of actual organ procurements, the aforementioned limitations are ones that the OPOs and transplant centers will need to address. Inability to transfer a patient will obviously prevent this scenario from being followed. Nevertheless, as more approaches are becoming available, the DCD heart donation may require a wide range of options to be available, and for those who are unwilling to transfer, the other available options can be exercised (eg, the use of MP by centers who will employ that particular approach). The cost of transfer of the potential donor has generally rested on the OPOs, but this model may need a shared cost model with the receiving hospital and will need to be worked out. Finally, in cases in which the potential donor does not progress to circulatory arrest, the same concerns that occur with or without NRP have been in play for many years, namely the requirement that the patient be transferred to an appropriate in-hospital setting for palliative care measures.

We recognize that as recent experience in the United States is expanding, different centers will be creative in their approach for implementing NRP. In one recent report, a modified ECMO system was used for distant procurement with static cold storage for transportation.^3^, ^4^ While that seems to be a plausible approach, the major limitations include the cost and personnel needed to send for each procurement, as well as the inability to fully evaluate the heart by more acceptable and standard tools currently available. One may argue that the “visual” assessment of the beating heart, which still remains a standard tradition, is itself insufficient in current times and that inadequate overall all assessment of heart function might portend graft failure and increased mortality, especially as this approach is expanded to nontraditional (ie, older) donors. This proposed regionalized approach could facilitate the expansion and coordination of DCD transplantation among many transplant centers and would have the potential to vastly expand access to quality DCD hearts, increase the pool of donors, and ultimately lead to a substantial growth in the number of heart transplants. This vision is only achievable, however, if heart transplant centers and their associated institutions are willing to join a multicenter network in a cooperative spirit and rely on each other to achieve this mission. We believe that the aforementioned approach is feasible and that by working closely with our respective OPOs, we can create DCD-dedicated procurement donor centers for the benefit of all patients. The onus is on us, but this vision does not mean that it will be the only way possible. In the end, as experience expands, this may be one good practical approach among many others.

### Conflict of Interest Statement

The authors reported no conflicts of interest.

The *Journal* policy requires editors and reviewers to disclose conflicts of interest and to decline handling or reviewing manuscripts for which they may have a conflict of interest. The editors and reviewers of this article have no conflicts of interest.
